# Supplementary material for: End Sequence Analysis Toolkit (ESAT) expands the extractable information from single-cell RNA-seq data
Source: Genome Res. 2016 Oct;26(10):1397–410. doi: 10.1101/gr.207902.116 (PMC5052061; doi:10.1101/gr.207902.116)
Supplement: Supplemental Material [file supp_gr.207902.116_Supp_Materials.tar.gz › ESAT-esat_v0.1/doc/html/classbroad_1_1core_1_1annotation_1_1_basic_lightweight_annotation.html]

ESAT: broad.core.annotation.BasicLightweightAnnotation Class Reference


ESAT


- Main Page
- Related Pages
- Packages
- Classes
- Files

- Class List
- Class Index
- Class Hierarchy
- Class Members

All Classes Namespaces Files Functions Variables Enumerator Pages

- broad
- core
- annotation
- BasicLightweightAnnotation

Public Member Functions |
Static Public Member Functions |
Protected Member Functions |
List of all members

broad.core.annotation.BasicLightweightAnnotation Class Reference

Inheritance diagram for broad.core.annotation.BasicLightweightAnnotation:

[legend]

Collaboration diagram for broad.core.annotation.BasicLightweightAnnotation:

[legend]

|  |  |
| --- | --- |
| Public Member Functions | |
|  | BasicLightweightAnnotation () |
|  | |
|  | BasicLightweightAnnotation (String chr, int start, int end) |
|  | |
|  | BasicLightweightAnnotation (String chr, int start, int end, String orientation) |
|  | |
|  | BasicLightweightAnnotation (String chr, int start, int end, Strand orientation, String name) |
|  | |
|  | BasicLightweightAnnotation (String chr, int start, int end, String orientation, double Scr) |
|  | |
|  | BasicLightweightAnnotation (String chr, String start, String end) |
|  | |
|  | BasicLightweightAnnotation (LightweightGenomicAnnotation annotation) |
|  | |
|  | BasicLightweightAnnotation (Annotation annotation) |
|  | |
| String | getChromosomeString () |
|  | |
| int | getDistanceTo (LightweightGenomicAnnotation other) |
|  | |
| double | getExtraScore (int i) |
|  | |
| List< Double > | getExtraScores () |
|  | |
| String | getLocationString () |
|  | |
| long | getMiddle () |
|  | |
| int | getOverlap (LightweightGenomicAnnotation other) |
|  | |
| double | getScore () |
|  | |
| void | setReversedOrientation (boolean isInReversedOrientation) |
|  | |
| void | setScore (double score) |
|  | |
| boolean | almostEqual (LightweightGenomicAnnotation other, int fudge) |
|  | |
| boolean | contains (LightweightGenomicAnnotation other) |
|  | |
| boolean | inReversedOrientation () |
|  | |
| boolean | overlaps (Collection<?extends Annotation > others, int buffer) |
|  | |
| boolean | overlaps (Collection<?extends Annotation > others) |
|  | |
| void | addExtraScore (double score) |
|  | |
| void | removeExtraScores () |
|  | |
| void | setBoundariesFromAnnoations (List<?extends GenomicAnnotation > annotations) |
|  | |
| void | stitchTo (LightweightGenomicAnnotation other) |
|  | |
| void | takeIntersection (LightweightGenomicAnnotation other) |
|  | |
| void | takeUnion (LightweightGenomicAnnotation other) |
|  | |
| LightweightGenomicAnnotation | intersect (LightweightGenomicAnnotation other) |
|  | |
| Collection<?extends Annotation > | intersect (Collection<?extends Annotation > annotations) |
|  | |
| int | hashCode () |
|  | |
| int | length () |
|  | |
| String | toUCSC () |
|  | |
| String | toString () |
|  | |
| int | compareTo (LightweightGenomicAnnotation b) |
|  | |
| void | setOrientation (String orientation) |
|  | |
| String | getChromosome () |
|  | |
| void | setChromosome (String chr) |
|  | |
| Public Member Functions inherited from broad.core.annotation.LightweightGenomicAnnotation | |
| int | getStart () |
|  | |
| int | getEnd () |
|  | |
| String | getName () |
|  | |
| void | setName (String name) |
|  | |
| Strand | getOrientation () |
|  | |
| void | setStart (int start) |
|  | |
| void | setEnd (int end) |
|  | |
| Public Member Functions inherited from umms.core.annotation.Annotation | |
| int | getSAMStart () |
|  | |
| int | getSAMEnd () |
|  | |
| String | getReferenceName () |
|  | |
| String | getChr () |
|  | |
| Strand | getStrand () |
|  | |
| boolean | hasOrientation () |
|  | |
| boolean | isNegativeStrand () |
|  | |
| int | numBlocks () |
|  | |
| List<?extends Annotation > | getBlocks () |
|  | |
| List<?extends Annotation > | getBlocks (boolean oriented) |
|  | |
| int | getSize () |
|  | |
| int | size () |
|  | |
| int | getLengthOnReference () |
|  | |
| int | getOrientedStart () |
|  | |
| int | getOrientedEnd () |
|  | |
| boolean | isUnoriented () |
|  | |
| int | getReferenceCoordinateAtPosition (int positionInAnnotation) |
|  | |
| int | getPositionAtReferenceCoordinate (int referenceCoordinate) |
|  | |
| int | getReferenceCoordinateAtPosition (int positionInAnnotation, boolean ignoreOrientation) |
|  | |
| int | getPositionAtReferenceCoordinate (int referenceCoordinate, boolean ignoreOrientation) |
|  | |
| void | setOrientation (char orientation) |
|  | |
| void | setOrientation (Strand orientation) |
|  | |
| void | setOrientedStart (int orientedStart) |
|  | |
| void | setOrientedEnd (int orientedEnd) |
|  | |
| void | setReferenceName (String refName) |
|  | |
| boolean | equals (Annotation other) |
|  | |
| void | expand (int deltaStart, int deltaEnd) |
|  | |
| Annotation | trim (int deltaStart, int deltaEnd) |
|  | |
| void | shift (int delta) |
|  | |
| void | moveToCoordinate (int coordinateInReference) |
|  | |
| Annotation | copy () |
|  | |
| List< Annotation > | disect (Annotation a) |
|  | |
| List< Annotation > | disect (List<?extends Annotation > disectors) |
|  | |
| Annotation | minus (Annotation other) |
|  | |
| Annotation | minus (Collection<?extends Annotation > others) |
|  | |
| int | getDistanceTo (Annotation other) |
|  | |
| String | toBED () |
|  | |
| String | toBED (int r, int g, int b) |
|  | |
| String | toShortBED () |
|  | |
| String | toBEDGraph () |
|  | |
| String | getFullInfoString () |
|  | |
| boolean | overlaps (Annotation other, int buffer) |
|  | |
| boolean | overlaps (Annotation other) |
|  | |
| boolean | overlaps (Annotation other, int buffer, boolean considerOrientation) |
|  | |
| boolean | overlaps (Annotation other, boolean considerOrientation) |
|  | |
| boolean | overlapsStranded (Annotation other) |
|  | |
| int | getOverlap (Annotation other) |
|  | |
| boolean | contains (Annotation other) |
|  | |
| Annotation | union (Annotation other) |
|  | |
| Annotation | intersect (Annotation other) |
|  | |
| List< Annotation > | intersect (List<?extends Annotation > annotations) |
|  | |
| int | compareToAnnotation (Annotation b) |
|  | |
| void | stitchTo (Annotation next) |
|  | |
| String | toCigar () |
|  | |
| boolean | fullyContains (Annotation annotation) |
|  | |
| Annotation | complement () |
|  | |
| boolean | equals (Annotation other, boolean useOrientation) |
|  | |
| Collection<?extends Annotation > | getSpliceConnections () |
|  | |
| int | getMidpoint () |
|  | |

|  |  |
| --- | --- |
| Static Public Member Functions | |
| static Collection  < BasicLightweightAnnotation > | mergeAllOverlappers (TreeSet< BasicLightweightAnnotation > regions) |
|  | |
| static LightweightGenomicAnnotation | createFromUCSC (String ucsc) |
|  | |
| static List< Annotation > | stitchList (Collection<?extends Annotation > sortedList, int maxDistanceToStitch) |
|  | |

|  |  |
| --- | --- |
| Protected Member Functions | |
| void | setStart (String data) |
|  | |

## Constructor & Destructor Documentation

|  |  |  |  |  |
| --- | --- | --- | --- | --- |
| broad.core.annotation.BasicLightweightAnnotation.BasicLightweightAnnotation | ( |  | ) |  |

Here is the caller graph for this function:

|  |  |  |  |
| --- | --- | --- | --- |
| broad.core.annotation.BasicLightweightAnnotation.BasicLightweightAnnotation | ( | String | *chr*, |
|  |  | int | *start*, |
|  |  | int | *end* |
|  | ) |  |  |

|  |  |  |  |
| --- | --- | --- | --- |
| broad.core.annotation.BasicLightweightAnnotation.BasicLightweightAnnotation | ( | String | *chr*, |
|  |  | int | *start*, |
|  |  | int | *end*, |
|  |  | String | *orientation* |
|  | ) |  |  |

|  |  |  |  |
| --- | --- | --- | --- |
| broad.core.annotation.BasicLightweightAnnotation.BasicLightweightAnnotation | ( | String | *chr*, |
|  |  | int | *start*, |
|  |  | int | *end*, |
|  |  | Strand | *orientation*, |
|  |  | String | *name* |
|  | ) |  |  |

|  |  |  |  |
| --- | --- | --- | --- |
| broad.core.annotation.BasicLightweightAnnotation.BasicLightweightAnnotation | ( | String | *chr*, |
|  |  | int | *start*, |
|  |  | int | *end*, |
|  |  | String | *orientation*, |
|  |  | double | *Scr* |
|  | ) |  |  |

|  |  |  |  |
| --- | --- | --- | --- |
| broad.core.annotation.BasicLightweightAnnotation.BasicLightweightAnnotation | ( | String | *chr*, |
|  |  | String | *start*, |
|  |  | String | *end* |
|  | ) |  |  |

|  |  |  |  |  |  |
| --- | --- | --- | --- | --- | --- |
| broad.core.annotation.BasicLightweightAnnotation.BasicLightweightAnnotation | ( | LightweightGenomicAnnotation | *annotation* | ) |  |

Here is the call graph for this function:

|  |  |  |  |  |  |
| --- | --- | --- | --- | --- | --- |
| broad.core.annotation.BasicLightweightAnnotation.BasicLightweightAnnotation | ( | Annotation | *annotation* | ) |  |

Here is the call graph for this function:

## Member Function Documentation

|  |  |  |  |  |  |
| --- | --- | --- | --- | --- | --- |
| void broad.core.annotation.BasicLightweightAnnotation.addExtraScore | ( | double | *score* | ) |  |

Adds a new extra score

Parameters
:   |  |  |
    | --- | --- |
    | score |  |

Implements broad.core.annotation.LightweightGenomicAnnotation.

Here is the caller graph for this function:

|  |  |  |  |
| --- | --- | --- | --- |
| boolean broad.core.annotation.BasicLightweightAnnotation.almostEqual | ( | LightweightGenomicAnnotation | *other*, |
|  |  | int | *fudge* |
|  | ) |  |  |

Checks whether two annotations differ by a small (fudge) factor

Parameters
:   |  |  |
    | --- | --- |
    | fudge | Maximum difference at either end or start to consider similar |

Returns

Implements broad.core.annotation.LightweightGenomicAnnotation.

Here is the call graph for this function:

|  |  |  |  |  |  |
| --- | --- | --- | --- | --- | --- |
| int broad.core.annotation.BasicLightweightAnnotation.compareTo | ( | LightweightGenomicAnnotation | *b* | ) |  |

Here is the call graph for this function:

|  |  |  |  |  |  |
| --- | --- | --- | --- | --- | --- |
| boolean broad.core.annotation.BasicLightweightAnnotation.contains | ( | LightweightGenomicAnnotation | *other* | ) |  |

Implements broad.core.annotation.LightweightGenomicAnnotation.

Here is the call graph for this function:

Here is the caller graph for this function:

|  |  |  |  |  |  |
| --- | --- | --- | --- | --- | --- |
| static LightweightGenomicAnnotation broad.core.annotation.BasicLightweightAnnotation.createFromUCSC | ( | String | *ucsc* | ) |  |

static

Here is the call graph for this function:

|  |  |  |  |  |
| --- | --- | --- | --- | --- |
| String broad.core.annotation.BasicLightweightAnnotation.getChromosome | ( |  | ) |  |

Implements broad.core.annotation.LightweightGenomicAnnotation.

Here is the call graph for this function:

Here is the caller graph for this function:

|  |  |  |  |  |
| --- | --- | --- | --- | --- |
| String broad.core.annotation.BasicLightweightAnnotation.getChromosomeString | ( |  | ) |  |

Returns the usual way to write a chromosome: chrSymbol if a chromosome or the full name of the scaffold

Implements broad.core.annotation.LightweightGenomicAnnotation.

Here is the call graph for this function:

Here is the caller graph for this function:

|  |  |  |  |  |  |
| --- | --- | --- | --- | --- | --- |
| int broad.core.annotation.BasicLightweightAnnotation.getDistanceTo | ( | LightweightGenomicAnnotation | *other* | ) |  |

public void

Implements broad.core.annotation.LightweightGenomicAnnotation.

Here is the call graph for this function:

|  |  |  |  |  |  |
| --- | --- | --- | --- | --- | --- |
| double broad.core.annotation.BasicLightweightAnnotation.getExtraScore | ( | int | *i* | ) |  |

Returns the ith extra score

Implements broad.core.annotation.LightweightGenomicAnnotation.

|  |  |  |  |  |
| --- | --- | --- | --- | --- |
| List<Double> broad.core.annotation.BasicLightweightAnnotation.getExtraScores | ( |  | ) |  |

Returns
:   all extra scores

Implements broad.core.annotation.LightweightGenomicAnnotation.

Here is the caller graph for this function:

|  |  |  |  |  |
| --- | --- | --- | --- | --- |
| String broad.core.annotation.BasicLightweightAnnotation.getLocationString | ( |  | ) |  |

Here is the call graph for this function:

Here is the caller graph for this function:

|  |  |  |  |  |
| --- | --- | --- | --- | --- |
| long broad.core.annotation.BasicLightweightAnnotation.getMiddle | ( |  | ) |  |

Implements broad.core.annotation.LightweightGenomicAnnotation.

Here is the call graph for this function:

|  |  |  |  |  |  |
| --- | --- | --- | --- | --- | --- |
| int broad.core.annotation.BasicLightweightAnnotation.getOverlap | ( | LightweightGenomicAnnotation | *other* | ) |  |

Parameters
:   |  |  |
    | --- | --- |
    | other | GenomicAnnotation to check of overlap |

Returns
:   true if the current instance overlaps with the other one. Returns the number of bases that overlap between the two annotations.

Parameters
:   |  |  |
    | --- | --- |
    | other |  |

Returns

Implements broad.core.annotation.LightweightGenomicAnnotation.

Here is the call graph for this function:

|  |  |  |  |  |
| --- | --- | --- | --- | --- |
| double broad.core.annotation.BasicLightweightAnnotation.getScore | ( |  | ) |  |

Implements broad.core.annotation.LightweightGenomicAnnotation.

Here is the caller graph for this function:

|  |  |  |  |  |
| --- | --- | --- | --- | --- |
| int broad.core.annotation.BasicLightweightAnnotation.hashCode | ( |  | ) |  |

Here is the call graph for this function:

|  |  |  |  |  |
| --- | --- | --- | --- | --- |
| boolean broad.core.annotation.BasicLightweightAnnotation.inReversedOrientation | ( |  | ) |  |

Implements broad.core.annotation.LightweightGenomicAnnotation.

Here is the call graph for this function:

Here is the caller graph for this function:

|  |  |  |  |  |  |
| --- | --- | --- | --- | --- | --- |
| LightweightGenomicAnnotation broad.core.annotation.BasicLightweightAnnotation.intersect | ( | LightweightGenomicAnnotation | *other* | ) |  |

Change the current instance to represent its intersection with the provided annotation if they overlap

Parameters
:   |  |  |
    | --- | --- |
    | other |  |

Here is the call graph for this function:

Here is the caller graph for this function:

|  |  |  |  |  |  |
| --- | --- | --- | --- | --- | --- |
| Collection<? extends Annotation> broad.core.annotation.BasicLightweightAnnotation.intersect | ( | Collection<?extends Annotation > | *annotations* | ) |  |

Here is the call graph for this function:

|  |  |  |  |  |
| --- | --- | --- | --- | --- |
| int broad.core.annotation.BasicLightweightAnnotation.length | ( |  | ) |  |

Returns
:   length of the Annotation; if it has blocks, the gaps between blocks are not included

Implements broad.core.annotation.LightweightGenomicAnnotation.

Here is the call graph for this function:

Here is the caller graph for this function:

|  |  |  |  |  |  |
| --- | --- | --- | --- | --- | --- |
| static Collection<BasicLightweightAnnotation> broad.core.annotation.BasicLightweightAnnotation.mergeAllOverlappers | ( | TreeSet< BasicLightweightAnnotation > | *regions* | ) |  |

static

Merge overlapping regions into a single region and leave singletons alone

Parameters
:   |  |  |
    | --- | --- |
    | regions | The regions to merge |

Returns
:   The set of merged regions

Here is the call graph for this function:

|  |  |  |  |
| --- | --- | --- | --- |
| boolean broad.core.annotation.BasicLightweightAnnotation.overlaps | ( | Collection<?extends Annotation > | *others*, |
|  |  | int | *buffer* |
|  | ) |  |  |

Parameters
:   |  |  |
    | --- | --- |
    | other | GenomicAnnotation to check of overlap |

Returns
:   true if the current instance overlaps with the other one.

Parameters
:   |  |  |
    | --- | --- |
    | other | - other genomic annotation |
    | buffer | if the overlap is within buffer they will be considered overlapping even if they do not overlap within their original boundaries. |

Returns
:   true if they overlap in this extended definition

Implements umms.core.annotation.Annotation.

Here is the caller graph for this function:

|  |  |  |  |  |  |
| --- | --- | --- | --- | --- | --- |
| boolean broad.core.annotation.BasicLightweightAnnotation.overlaps | ( | Collection<?extends Annotation > | *others* | ) |  |

Parameters
:   |  |  |
    | --- | --- |
    | other | GenomicAnnotations to check of overlap |

Returns
:   true if the current instance overlaps with the other one, not considering orientation

Implements umms.core.annotation.Annotation.

Here is the call graph for this function:

|  |  |  |  |  |
| --- | --- | --- | --- | --- |
| void broad.core.annotation.BasicLightweightAnnotation.removeExtraScores | ( |  | ) |  |

removes extra scores

Implements broad.core.annotation.LightweightGenomicAnnotation.

|  |  |  |  |  |  |
| --- | --- | --- | --- | --- | --- |
| void broad.core.annotation.BasicLightweightAnnotation.setBoundariesFromAnnoations | ( | List<?extends GenomicAnnotation > | *annotations* | ) |  |

Here is the call graph for this function:

|  |  |  |  |  |  |
| --- | --- | --- | --- | --- | --- |
| void broad.core.annotation.BasicLightweightAnnotation.setChromosome | ( | String | *chr* | ) |  |

Implements broad.core.annotation.LightweightGenomicAnnotation.

Here is the call graph for this function:

Here is the caller graph for this function:

|  |  |  |  |  |  |
| --- | --- | --- | --- | --- | --- |
| void broad.core.annotation.BasicLightweightAnnotation.setOrientation | ( | String | *orientation* | ) |  |

Implements broad.core.annotation.LightweightGenomicAnnotation.

Here is the caller graph for this function:

|  |  |  |  |  |  |
| --- | --- | --- | --- | --- | --- |
| void broad.core.annotation.BasicLightweightAnnotation.setReversedOrientation | ( | boolean | *isInReversedOrientation* | ) |  |

Here is the call graph for this function:

Here is the caller graph for this function:

|  |  |  |  |  |  |
| --- | --- | --- | --- | --- | --- |
| void broad.core.annotation.BasicLightweightAnnotation.setScore | ( | double | *score* | ) |  |

Implements broad.core.annotation.LightweightGenomicAnnotation.

Here is the caller graph for this function:

|  |  |  |  |  |  |
| --- | --- | --- | --- | --- | --- |
| void broad.core.annotation.BasicLightweightAnnotation.setStart | ( | String | *data* | ) |  |

protected

Here is the caller graph for this function:

|  |  |  |  |
| --- | --- | --- | --- |
| static List<Annotation> broad.core.annotation.BasicLightweightAnnotation.stitchList | ( | Collection<?extends Annotation > | *sortedList*, |
|  |  | int | *maxDistanceToStitch* |
|  | ) |  |  |

static

Parameters
:   |  |  |
    | --- | --- |
    | sortedList |  |
    | maxDistanceToStitch |  |

Returns

Here is the call graph for this function:

Here is the caller graph for this function:

|  |  |  |  |  |  |
| --- | --- | --- | --- | --- | --- |
| void broad.core.annotation.BasicLightweightAnnotation.stitchTo | ( | LightweightGenomicAnnotation | *other* | ) |  |

Enlarges this annotation by

Parameters
:   |  |  |
    | --- | --- |
    | other |  |

Implements broad.core.annotation.LightweightGenomicAnnotation.

Here is the call graph for this function:

|  |  |  |  |  |  |
| --- | --- | --- | --- | --- | --- |
| void broad.core.annotation.BasicLightweightAnnotation.takeIntersection | ( | LightweightGenomicAnnotation | *other* | ) |  |

Change the current instance to represent its intersection with the provided annotation if they overlap

Parameters
:   |  |  |
    | --- | --- |
    | other |  |

Implements broad.core.annotation.LightweightGenomicAnnotation.

Here is the call graph for this function:

|  |  |  |  |  |  |
| --- | --- | --- | --- | --- | --- |
| void broad.core.annotation.BasicLightweightAnnotation.takeUnion | ( | LightweightGenomicAnnotation | *other* | ) |  |

Change the current instance to represent its union with the provided annotation

Parameters
:   |  |  |
    | --- | --- |
    | GenomicAnnotation | other |

Implements broad.core.annotation.LightweightGenomicAnnotation.

Here is the call graph for this function:

|  |  |  |  |  |
| --- | --- | --- | --- | --- |
| String broad.core.annotation.BasicLightweightAnnotation.toString | ( |  | ) |  |

Here is the call graph for this function:

|  |  |  |  |  |
| --- | --- | --- | --- | --- |
| String broad.core.annotation.BasicLightweightAnnotation.toUCSC | ( |  | ) |  |

Similar to getLocationString()

See also
:   getLocationString and provided only for backwards compatibility.

Implements broad.core.annotation.LightweightGenomicAnnotation.

Here is the call graph for this function:

Here is the caller graph for this function:

---

The documentation for this class was generated from the following file:

- java/broad/core/annotation/BasicLightweightAnnotation.java


---

Generated on Fri Aug 15 2014 15:10:50 for ESAT by  

 1.8.7
